# A Comparison of the Effects of Phycocyanin, γ-Aminobutyric Acid, Glycine Betaine, and Mycorrhizal Biostimulants of Non-Stressed *Agrostis stolonifera*

**DOI:** 10.3390/plants14142110

**Published:** 2025-07-09

**Authors:** Iván Darío Samur Suárez, Tom Hsiang, Paul H. Goodwin

**Affiliations:** School of Environmental Sciences, University of Guelph, 50 Stone Road East, Guelph, ON N1G 2W1, Canada; samurivandario@gmail.com (I.D.S.S.); thsiang@uoguelph.ca (T.H.)

**Keywords:** biomass, cultivar, dry weight, fresh weight, greeness, greenhouse, *Rhizophagus intraradices*

## Abstract

Four biostimulants (phycocyanin, γ-aminobutyric acid (GABA), glycine betaine (GB), and the mycorrhizal fungus *Rhizophagus intraradices*) were applied foliarly to six cultivars of mature creeping bentgrass (*Agrostis stolonifera*) under non-stressed greenhouse conditions. Phycocyanin was most effective at increasing total shoot greenness, which was most consistent over time with the cultivars Penncross, T1, and Tyee. GABA was most effective at increasing total root fresh and dry weight, most strongly for Penncross and T1, respectively. GB was most effective at increasing total shoot fresh and dry weight, with both most strongly increased for Tyee. By comparison, *R. intraradices* had relatively low effectiveness for increasing any of these parameters. The appearance of the grass at the end of the experiment revealed that 007 and Focus generally showed the most and least growth benefit, respectively, with all four biostimulants. However, all cultivars showed increases in more than one parameter for each biostimulant, and thus, no cultivar was uniformly responsive or non-responsive to all the biostimulants. This research shows that phycocyanin, GABA, and GB may benefit multiple creeping bentgrass cultivars under non-stressed conditions, but each one tended to be more beneficial to a particular aspect of plant growth and quality. End users need to be aware of the importance of creeping bentgrass genotype when considering biostimulant application.

## 1. Introduction

Perennial turfgrasses are used world-wide for recreation, farming, and habitat conservation [1]. Healthy turfgrass provides numerous ecosystem benefits, such as preventing soil erosion, sequestering carbon, regulating temperature, and reducing noise [2]. Turfgrasses also have a social value to improve human health by providing aethestics in parks, sport fields, roadsides, and at the edges of water ways [3]. However, they can require significant amounts of input, such as nitrogen fertilization, irrigation, and pesticides [4]. In the cool-season regions of North America, creeping bentgrass (*Agrostis stolonifera*) is a widely used turfgrass, particularly for putting greens on golf courses, which require intense management to maintain low mowing heights and high quality.

Biostimulants are compounds that increase plant growth and quality when applied in small amounts to foliage or soil under favorable conditions, and increase their biotic and abiotic stress resistance/tolerance under unfavorable conditions [5]. Biostimulants positively impact plant metabolism by enhancing nutritional efficiency, stress tolerance, and other functions, but they do not act directly as plant fertilizers [6]. du Jardin [6] listed seven categories of plant biostimulants, based on their source material: inorganic compounds, humic/fulvic acid, protein hydrolysates/N-containing compounds, seaweed extracts/botanicals, chitosan/biopolymers, beneficial fungi, and beneficial bacteria. However, Yakhin et al. [5] limited the definition of biostimulants to products of biological origin, whose benefits are not solely a consequence of the presence of known essential plant nutrients, plant growth regulators, or plant protective compounds.

For many crops, biostimulants are environmentally friendly alternatives to chemical inputs to address plant production issues such as sustainability [7,8]. Schmidt et al. [9] noted that the use of biostimulants has been promoted as a management practice in the turfgrass industry to increase grass chlorophyll content, photosynthesis, shoot–root ratio, and metabolism. A review of the use of biostiumulants in turfgrasses noted examples of humic and fulvic acids, seaweed extracts, protein hydrolysates, free amino acids, chitosan, and various fungi among those tested on turfgrasses [10].

The goal of this work was to test biostimulants for their ability to improve growth and quality of creeping bentgrass under low stress conditions (lack of wear stress, no drought stress, and adequate nitrogen), as improvements to turfgrass under non-stressed conditions are important and less often examined. A preliminary screening of various biostimulants on seedlings of creeping bentgrass cv Penncross showed that application of phycocyanin, γ-aminobutyric acid (GABA), glycine betaine (GB), and the mycorrhizal fungus *Rhizophagus intraradices* (syn. *R. irregularis*, *Glomus intraradices*) resulted in beneficial effects. Several of these biostimulants have previously been shown to improve the growth and quality of stressed turfgrasses. For example, GABA application alleviated heat stress [11] and drought stress [12] in creeping bentgrass, GB treatment of six turfgrass species enhanced tolerance to salinity, drought, and temperature stress [13], and a mixture of the mycorrhizal fungi *Funneliformis mosseae* and *R. irregularis* resulted in higher above- and below-ground weights and greater visual greenness of two turfgrass species under drought stress [14]. The use of phycocyanin on turfgrass has not been reported, but soaking tomato seeds with a phycocyanin extract resulted in seedlings developing significantly greater weight and length, as well as total carbohydrate and protein contents, compared with a non-treated control [15]. The objective of this study was to determine the effects of phycocyanin, GABA, GB, and *R. intraradices* on the greenness and biomass of six cultivars of mature creeping bentgrass grown in the greenhouse, in replicated experiments, and to determine whether the cultivar affected the response. While the cultivars had an effect, no cultivar was responsive to all the biostimulants tested. Thus, turfgrass managers should consider the plant cultivar prior to application.

## 2. Results

### 2.1. Effect of Foliar Phycocyanin on Greenness and Biomass of Six Creeping Bentgrass Cultivars

**Figure 1:**
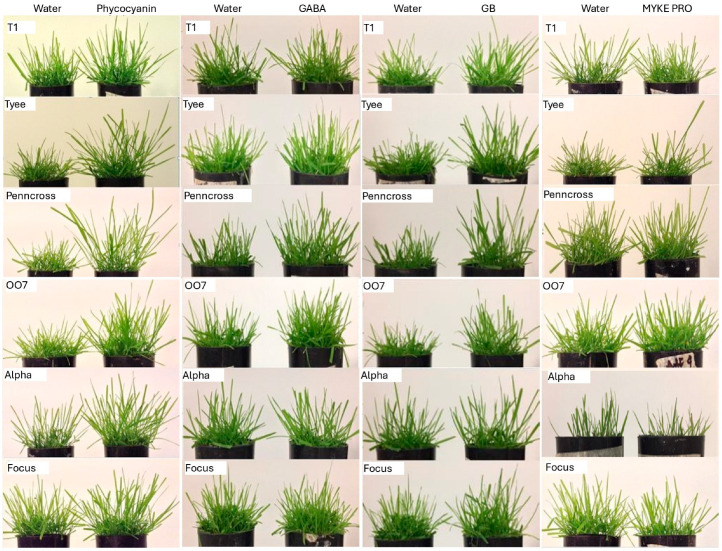
Appearance of plugs of creeping bentgrass (*A. stolonifera*) cultivars T1, Tyee, Penncross, 007, Alpha, and Focus with foliar water (control) versus treatment with phycocyanin, γ-aminobutyric acid (GABA), glycine betaine (GB), or *Rhizophagus intraradices* (MYKE PRO) at 28 DPT.

Compared with the water control, phycocyanin significantly increased the greenness index of clippings for all cultivars tested at 7 DPT (days post treatment), and all cultivars, except Alpha at 14 DPT, Focus at 21 DPT, and 007 at 28 DPT (Table 1). Penncross, T1, and Tyee cultivars showed significant increases in greenness with phycocyanin at all time points. The# fresh weight of clippings was significantly higher with phycocyanin for all cultivars, except Penncross and T1 at 7 DPT and Alpha at 14 DPT, but it was significantly higher with phycocyanin for all cultivars at 21 DPT (Table 2). The dry weight of clippings was significantly increased with phycocyanin for all cultivars, except T1 at 7 DPT, Alpha at 14 DPT, but was significantly higher for all cultivars at 21 DPT. The only cultivars to show both significantly higher fresh and dry weights of clippings at all time points with phycocyanin were Focus and 007. At 28 DPT, total shoot fresh weight was significantly increased with phycocyanin for all cultivars, but total shoot dry weight was significantly increased only for Alpha, Penncross, and T1 (Table 3). Only Alpha, Penncross, and T1 had significantly higher fresh and dry shoot weights. At 28 DPT, total root fresh weight was significantly increased with phycocyanin only for 007 and Tyee, whereas total root dry weight was significantly increased for all cultivars except Alpha. Only 007 and Tyee had both significantly higher fresh and dry root weights. By 28 DPT, the difference between the appearance of plugs of water and phycocyanin treated plants was greatest for Tyee, Penncross, and 007 with notably more growth, although all cultivars showed visible growth responses (Figure 1). Thus, a consistently higher weekly greenness index with phycocyanin treatment for each cultivar did not correlate with consistently higher weekly fresh and dry weights of clippings. However, a consistently increased greenness index due to phycocyanin corresponded to both higher fresh and dry shoot weights for Penncross and T1, and it corresponded to both higher fresh and dry root weights for Tyee at the end of the experiment.

### 2.2. Effect of Foliar γ-Aminobutyric Acid on Greenness and Biomass of Six Creeping Bentgrass Cultivars

GABA significantly increased the greenness index for Alpha at 7 DPT, Focus, 007, and Tyee at 14 DPT, all cultivars at 21 DPT, and Focus and Penncross at 28 DPT (Table 4). While Focus most often had significantly higher greenness due to GABA, no cultivar showed significantly higher greenness at all time points. GABA significantly increased the fresh weight of clippings for 007 and Penncross at 7 DPT, Tyee at 14 DPT, and Alpha and T1 at 21 DPT (Table 5). The dry weight of clippings was not significantly increased by GABA for any cultivar at 7 or 14 DPT, but for Alpha, Focus, Penncross, and T1 at 21 DPT. No cultivar showed both significantly higher fresh and dry weights of clippings at all or even most time points. At 28 DPT, GABA resulted in total shoot fresh weight that was significantly increased for all cultivars, and it resulted in total shoot dry weight that was significantly increased for all cultivars, except Tyee (Table 6). At 28 PDT, total root fresh weight was significantly increased by GABA for all cultivars, and total root dry weight was significantly increased by GABA for all cultivars, except Focus and Tyee. The difference in the appearance of plants between water and GABA at 28 DPT was most visible for Penncross and 007 (Figure 1). Thus, although the consistent effects of GABA on greenness and clipping biomass over time were relatively slight, it was effective in increasing total biomass for most of the cultivars, particularly root fresh and dry weights, except for Focus and Tyee, by the end of the experiment.

### 2.3. Effect of Foliar Glycine Betaine on Greenness and Biomass of Six Creeping Bentgrass Cultivars

GB application significantly increased the greenness index for all cultivars except Penncross and T1 at 7 DPT, 007 and T1 at 14 DPT, all cultivars at 21 DPT, and all cultivars except Alpha at 28 DPT (Table 7). Only 007 showed a consistent response with significant increases at all time points. For fresh weight of clippings, GB resulted in significant increases for 007 at 7 DPT, all cultivars except Focus and Penncross at 14 DPT, and all cultivars at 21 DPT, while dry weights of clippings showed significant increases with GB for none of the cultivars at 7 DPT, 007 and Tyee at 14 DPT, and all cultivars except T1 at 21 DPT (Table 8). Cultivar 007 most commonly showed an increase in both fresh and dry weight of clippings over time, but no cultivar had significant increases in those parameters at all time points. For total shoot fresh weight at 28 DPT, significant increases were observed with GB for all cultivars, whereas total shoot dry weight at 28 DPT was significantly increased with GB for all cultivars, except Focus (Table 9). GB resulted in significant increases in both total root fresh and dry weights for all cultivars at 28 DPT. Based on appearance, Alpha and Focus showed the least difference between water and GB treatment, whereas Tyee, Penncross, and 007 showed the greatest differences (Figure 1). Thus, GB was inconsistent in increasing greenness over time, while biomass of clippings increased in most cultivars after several applications correlating with increased total biomass at the end of the experiment for almost all cultivars, most strongly with shoot fresh and dry weights.

### 2.4. Effect of Foliar Rhizophagus Intraradices on Greenness and Biomass of Six Creeping Bentgrass Cultivars

MYKE PRO significantly increased greenness for all cultivars except 007 at 7 DPT, Alpha, 007, and Penncross at 14 DPT, T1 and Tyee at 21 DPT, and all cultivars except Focus and 007 at 28 DPT (Table 10). While Penncross, T1, and Tyee most often had increased greenness with MYKE PRO, no cultivar had significant increases in greenness index at all time points. Regarding fresh weight of clippings, MYKE PRO resulted in significant increases for Alpha, 007, and Penncross at 7 DPT, 007 at 14 DPT, and Tyee at 21 DPT, while for dry weight of clippings, MYKE PRO resulted in significant increases for all cultivars except Tyee at 7 DPT, all cultivars except Focus and Tyee at 14 DPT, but no cultivars at 21 DPT (Table 11). While cultivar 007 was relatively more responsive to MYKE PRO in terms of both fresh and dry weight of clippings, no cultivar had significant increases at all time points. There were no cultivars with significantly increased total shoot fresh weights at 28 DPT with MYKE PRO, but Alpha, Focus, and 007 had significant increases with MYKE PRO for total shoot dry weight at 28 DPT (Table 12). For total root fresh weight at 28 DPT, no cultivars showed significant increases with MYKE PRO, but Alpha, 007, T1, and Tyee had significant increases in total root dry weights. Not only did no cultivar show consistent increases in total shoot and root fresh weights with MYKE PRO at 28 DPT, total shoot and root fresh weights were lower for all cultivars with MYKE PRO compared with the control. However, there were several cases of increased total shoot and root dry weights at 28 DPT with MYKE PRO, with Alpha and 007 experiencing the most consistent increases, indicating that the lower fresh weights may have been due to lower plant water contents with MYKE PRO. The appearance of plants treated with MYKE PRO was quite similar to the controls, although T1, 007, and Alpha appeared bushier, while Focus appeared less bushy (Figure 1). MYKE PRO treatment was the least beneficial among the biostimulants tested in this study, resulting in only occasional significant increases in greenness, clipping biomass, and total shoot and root biomass, with 007 and Alpha more often showing increases.

## 3. Discussion

### 3.1. Introduction

There is considerable evidence that biostimulants’ effectiveness can be impacted by the genotype of a plant. For example, the effects of four commercial biostimulant products differed in their effects on the chemical composition of the Fuji F1 versus the Viroflay genotypes of spinach [16], three commercial biostimulant products differed in their effects on specific leaf area and chlorophyll content between the very early potato cultivars Denar, Lord, and Milek [17], and the effects of two commercial biostimulant products on potato leaf chorophyll content, plant height, yield, and tuber quality differed significantly between potato cultivars Désirée, Kennebec, and Spunta [18]. However, the genotype effect has not been examined with biostimulants on turfgrasses, although the effects of seaweed extracts on yield and various leaf components have been shown to be affected by the genotype of certain pasture grasses [10]. In this study, there were clearly some differences between the six creeping bentgrass cultivars in their response to the biostimulants, but the amounts differed for each tested biostimulant.

### 3.2. Phycocyanin

Foliar application of phycocyanin was one of the most consistent biostimulants for increasing the greenness index of leaves over time, particularly for Penncross, T1, and Tyee. However, the effect appears to have taken time to translate into increased leaf biomass, as it was at 21 and 28 DPT that leaf fresh weights were increased for all cultivars in terms of clippings or total shoots, respectively. These benefits are consistent with Varia et al. [19], who reported that phycocyanin application increased both lettuce leaf greenness and yield. Phycocyanin is a pigment protein belonging to the light-harvesting phycobiliprotein family [20]. Among the phycobiliproteins, phycocyanin is the main light harvesting pigment in cyanobacteria (blue-green algae) and red algae, and cyanobacteria are often used for phycocyanin extraction as they contain relatively high amounts of the compound [21]. The key precursor of phycocyanin is 5-aminolevulinic acid (ALA), and ALA application can improve plant growth and yield, such as for kidney bean, potato, garlic, and radish [22,23]. The ALA plant growth-promoting effect is often attributed to the accumulation of phycocyanin and chlorophyll intracellularly in the plants, thus improving their photosynthetic activity [24]. A significant increase in photosynthetic rate was observed when ALA was applied to the foliage of 2-year-old grapevines, which could be related to the accumulation of phycocyanin [25]. Exogenously applied phycocyanin improved the photosynthetic efficiency of lettuce, perhaps acting on the glycolate pathway or even contributing to the plant’s core photosynthetic process since the addition of functional cyanobacterial components into plant chloroplasts improves photosynthetic efficiency [26]. However, phycocyanin has other properties, such as biosurfactant activity [27], that could also improve plant growth. There has been relatively little study of extracted phycocyanin as a biostimulant, but the current study showed that it clearly benefited multiple cultivars of creeping bentgrass, particularly for increasing leaf greenness.

### 3.3. γ-Aminobutyric Acid

Foliar application of GABA was inconsistent in significantly improving the greenness and biomass of leaf clippings over time, for all cultivars. However, increased total fresh weights of shoots and roots were observed with GABA for all cultivars, as well as increased total dry weights of shoots and roots for most cultivars by the end of the experiment, indicating plant growth stimulation that was mostly independent of the genotypes tested. The increases in shoot and root fresh but not dry weights indicated that GABA may have increased the water content in some cultivars. GABA is a non-protein amino acid that can accumulate rapidly when a plant is exposed to abiotic stresses regulating stress responses as well as growth and development [28]. There have been several studies applying GABA to drought-, salt-, and temperature-stressed turfgrasses, showing that it can enhance photosynthesis, antioxidant defenses, membrane integrity, osmotic levels, and ion hemostasis under stress [29]. For example, exogenous foliar application of GABA on heat-stressed creeping bentgrass increased greenness and photosynthetic rate [11]. Exogenous GABA improved the heat and drought stress tolerance of creeping bentgrass associated with increased leaf water content, cell membrane stability, chlorophyll content and transcript levels of genes for stress-protective proteins and transcription factors related to heat and drought tolerance [30]. Exogenous GABA application to creeping bentgrass also improved salt tolerance that was associated with reduced stomatal conductance, increased photosynthesis, stronger osmotic adjustment, greater water use efficiency, and upregulated expression of genes involved in mitigation of sodium toxicity [31]. Thus, there is considerable evidence that GABA can increase stress tolerance of creeping bentgrass associated with many changes in the physiology of the plant. However, this is the first report to show that GABA can have beneficial effects on turfgrass under non-stressed conditions, although this has been reported for other plants, such as non-stressed maize seedlings grown in pots under controlled conditions [32].

### 3.4. Glycine Betaine

For all cultivars, foliar exogenous GB was also relatively inconsistent in increasing greenness and leaf clipping biomass over time, although 007 most commonly showed an increase. Similar to phycocyanin, it may have taken multiple GB applications and time to impact the plants, as its effects on clipping greenness and fresh weight were mostly observed at 21 DPT and on total shoot and root fresh and dry weights at 28 DPT. GB is synthesized in chloroplasts from serine via ethanolamine, choline, and betaine aldehyde, and it accumulates in response to water stress, mainly in chloroplasts protecting thylakoid membranes, to maintain photosynthetic efficiency, thus providing protection against drought, salt, high temperature, and low temperature stresses [33]. For example, under drought stress conditions, foliar applications of GB enhanced the quality and Normalized Difference Vegetation Index (NDVI) of creeping bentgrass [34]. Clippings from drought-stressed perennial ryegrass also had significantly higher nitrogen, sulfur, potassium, and chloride contents with foliar GB treatment [35]. Exogenous application of GB to perennial ryegrass increased cadmium tolerance with increased turf quality, vertical shoot growth rate, transpiration, chlorophyll content, antioxidant enzyme activity, and gene expression, with decreased cadmium accumulation in both shoots and roots [36]. Similarly, GB application increased the salt tolerance of perennial ryegrass, which was associated with increased vertical shoot growth rate, relative water content, transpiration, chlorophyll content, potassium content, and antioxidative enzyme activity, but it decreased Na^+^ accumulation, resulting in a higher potassium/sodium ratio under saline conditions [37]. However, GB application has been reported to be ineffective on non-stressed turfgrasses. For example, GB-treated control perennial ryegrass (i.e., the non-stressed plants) did not cause significant increases in turf quality, vertical shoot growth rate, transpiration, chlorophyll content, or antioxidant enzyme activity and gene expression [36]. Also, for non-stressed perennial ryegrass, foliar applications of 50 mM GB did not significantly increase shoot or root fresh weight or chlorophyll content [37]. However, this study showed that foliar GB application can benefit creeping bentgrass under non-stressed conditions.

### 3.5. Rhizophagus intraradices

The *R. intraradices* inoculant MYKE PRO increased greenness and the weight of the clippings, but not for all cultivars, and the effects were not consistent over time for each cultivar. It did not increase total shoot and root fresh weight for any cultivar. Cultivars Alpha and 007 may have been the most responsive to MYKE PRO as they showed both increased shoot and root dry weight. *Rhizophagus intraradices* is an arbuscular mycorrhizal fungus (AMF), which are a group of obligate biotrophs forming mutualistic symbioses with roots of plants belonging to all the major land plant taxa [38]. Infected roots have extraradical mycelium extending into the soil that can absorb and transfer water and minerals, and AMF can increase the tolerance of plants to biotic and abiotic stresses as well as produce plant growth-promoting compounds, such as indole acetic acid, siderophores, and antibiotics [38] (Giovannini et al., 2020). For turfgrass, inoculation of a mixture of Kentucky bluegrass, red fescue, and perennial ryegrass at seeding time with *G. intraradices* resulted in faster coverage with no irrigation or fertilizer inputs [39], and drought-stressed tall fescue inoculated with *R. irregularis* showed increased shoot phosphorus, drought-response solutes (proline and glycine betaine), and antioxidant enzymes (superoxide dismutase and peroxidase) [40]. Also, several types of cadmium-stressed turfgrasses had less hydrogen peroxide and lipid peroxidation along with increased catalase, peroxidase, and superoxide dismutase activities with *R. irregularis* treatment [41]. This is the first report of *R. irregularis* enhancing turfgrass growth under non-stressed conditions, although some other plant species have shown improved growth with *R. irregularis* under non-stressed conditions [42]. Overall, *R. intraradices* was the least effective among the biostimulants tested in this study.

### 3.6. Comparisons and Outcomes

Plant biostimulants have multifaceted impacts on plants, with many direct effects such as stimulating carbon and nitrogen metabolism, as well as many indirect effects such as improving nutrient uptake and altering root morphology [43]. The different biostimulants tested in this study probably had different biochemical effects on non-stressed turfgrass, thus the degree of impact differed depending upon the parameter measured. Summarizing across all six creeping bentgrass cultivars examined, phycocyanin was most effective at increasing total shoot greenness showing an average 11% increase, GABA was most effective at increasing total root fresh and dry weight, with 35% and 32% increases, respectively, and GB was most effective at increasing total shoot fresh and dry weight, with 38% and 26% increases, respectively. As phycocyanin can improve photosynthetic efficiency [26], it is possible that some creeping bentgrass cultivars are better able to incorporate it into their chlorophyll, resulting in increased greeness. Improved plant growth with GABA is associated with higher cellular water retention, better cell membrane stability, and increased levels of proteins related to stress protection [30], and therefore, the roots of some creeping bentgrass cultivars may have been better able to utilize it and obtain these benefits even without stress. GB protects thylakoid membranes promoting photosynthetic efficiency [33], and some creeping bentgrass cultivars may have chloroplasts that incorporate it better, promoting photosynthesis and resulting in more above-ground growth, even in the absence of stress. MYKE PRO was not the most effective in increasing any of the parameters measured, possibly because the roots did not sufficiently colonize the roots or trigger an effective mutualistic symbiosis. While this study has demonstrated that several biostimulants can improve both growth and quality of a non-stressed turfgrass species, future research is needed to examine the mechanisms by which each one acts and how the host genotype can affect those modes of action.

## 4. Materials and Methods

### 4.1. Biological Materials

Plugs (3.5 cm wide × 12 cm deep) of mature *A. stolonifera* cultivars ‘Alpha, Focus, T1, Tyee, Penncross and 007’ were randomly selected from 20 m × 20 m swards of 5-year-old established plots at the Guelph Turfgrass Institute (GTI). These were transplanted into black plastic conetainers (3.8 cm diameter × 14 cm depth; SC7R Cell, Ray Leach Conetainer, Stuewe and Sons, Tangent, OR, USA) that were held in trays (RL98 tray with 98 cells, Stuewe and Sons, Tangent, OR, USA). Prior to treatment, the plants were established in a greenhouse for 3 weeks at 25 °C with regular watering and fertilization and then grown under the same conditions during and after treatment. The plants were trimmed weekly to 2 cm height.

### 4.2. Treatments

After each weekly trimming to 2 cm height, plants were treated with 3 mL of phycocyanin (CN Lab Nutrition, Asian Group, North York, ON, Canada) (7.6 mg/mL), γ-aminobutyric acid (GABA) (Sigma Aldrich, Oakville, ON, Canada) (0.016 mg/mL), glycine betaine (GB) (Sigma Aldrich) (0.52 mg/mL) and powdered mycorrhizal inoculum of *R. intraradices* (MYKE PRO) (Premier Tech Biotechnologies, Rivière-du-Loup, QC, Canada) (0.30 mg/mL) until runoff. Control plants were sprayed with 3 mL of sterile deionized water. Foliar application was carried out at 0, 7, 14, and 21 DPT. The experiment was repeated three times, with five containers for each cultivar per treatment in each experiment.

### 4.3. Assessments

The greenness, fresh weight, and dry weight of the clippings were assessed on 0, 7, 14, and 21 DPT. At 28 DPT, the soil was removed, and total shoot and root fresh and dry weights were determined. Fresh weight was determined immediately after harvesting, and dry weight was determined after incubating the tissues at 60 °C for 72 h. The greenness index as an indicator of canopy greenness was assessed using an iPad (Model A1432) (Apple Inc., Cupertino, CA, USA) with FieldScout GreenIndex + Turf (Spectrum Technologies Inc., Aurora, IL, USA) version 2.0 app for Apple mobile devices, which quantified the dark green color index (DGCI) of the leaves. This software measures the greenness of plants by analyzing the red, green, and blue (RGB) colors in a picture. RGB values are transformed into hue, saturation, and brightness values [44]. DGCI = [(hue − 60)/60 + (1 − saturation) + (1 − brightness)]/3.

### 4.4. Statistical Analysis

Statistical analyses were conducted using PROC GLM in SAS 9.1 (SAS Institute Inc., Cary, NC, USA; 2018) to test the significance of main effects and the first order interactions. When significant treatment effects were found, means were compared with Fisher’s protected least significant differences test at *p* < 0.05.

## 5. Conclusions

Much of the work on biostimulants in turfgrasses has concentrated on their effects of relieving stress conditions on the plants. In this study, foliar application of four different biostimulants resulted in statistically significant improvements in the growth and quality of creeping bentgrass, even under non-stressed conditions, but each biostimulant differed in terms of which plant parameter was most affected. Although all biostimulants affected more than one cultivar, this study revealed that the effectiveness of each biostimulant was influenced by the creeping bentgrass genotype. This should be considered when they are being applied to turfgrasses, which can be overseeded over time resulting in a mixture of different cultivars and species, thus affecting the effectiveness of biostimulants. The ability to increase both the quality and the growth of multiple cultivars of established non-stressed creeping bentgrass plants shows that phycocyanin, γ-aminobutyric acid, glycine betaine, and *R. intraradices* have considerable potential as biostimulants to provide more environmentally friendly alternatives to the many chemical inputs often used on turfgrasses. Future research could examine the underlying mechanisms of each biostimulant, such as stress-responsive gene expression and photosynthetic efficiency, to determine which mechanisms are related to improved growth and quality of creeping bentgrass, as well as testing these biostimulants in the field.

## Figures and Tables

**Table 1:** Average greenness index with weekly foliar application of phycocyanin for different cultivars of creeping bentgrass (*A. stolonifera*) at 7 to 28 DPT in the greenhouse.

Cultivar	Greenness Index ^a^							
	7 DPT		14 DPT		21 DPT		28 DPT	
	Phycocyanin ^b^	Water	Phycocyanin	Water	Phycocyanin	Water	Phycocyanin	Water
Alpha	0.607 *	0.565	0.585	0.585	0.578 *	0.502	0.554 *	0.490
Focus	0.597 *	0.548	0.584 *	0.543	0.502	0.518	0.549 *	0.501
007	0.591 *	0.552	0.570 *	0.546	0.567 *	0.514	0.540	0.492
Penncross	0.596 *	0.561	0.588 *	0.545	0.570 *	0.505	0.562 *	0.490
T1	0.590 *	0.539	0.584 *	0.548	0.550 *	0.517	0.545 *	0.503
Tyee	0.590 *	0.550	0.587 *	0.552	0.567 *	0.508	0.551 *	0.495

^a^ Greenness index values (0 = low to 1 = high) were obtained using FieldScout GreenIndex + Turf Spectrum Technologies, Inc. (Aurora, IL, USA) version 2.0 app. ^b^ Phycocyanin (7.6 mg/mL) applied to foliage at 0, 7, 14 and 21 DPT. * Significant difference between treatment and water control based on *t*-tests comparing average greenness index values from 15 replicates over 3 experiments (*p* < 0.05).

**Table 2:** Average clipping biomass with weekly foliar application of phycocyanin for different cultivars of creeping bentgrass (*A. stolonifera*) at 7 to 21 DPT in the greenhouse.

Cultivar	7 DPT		14 DPT		21 DPT	
	Fresh Weight (mg) ^a^					
	Phycocyanin ^b^	Water	Phycocyanin	Water	Phycocyanin	Water
Alpha	0.118 *	0.087	0.132	0.132	0.079 *	0.069
Focus	0.112 *	0.095	0.151 *	0.092	0.079 *	0.041
007	0.095 *	0.078	0.130 *	0.086	0.068 *	0.040
Penncross	0.103	0.100	0.125 *	0.076	0.084 *	0.036
T1	0.109	0.106	0.128 *	0.096	0.076 *	0.044
Tyee	0.087 *	0.067	0.132 *	0.078	0.068 *	0.040
	**Dry Weight (mg) ^a^**					
Alpha	0.027 *	0.021	0.026	0.026	0.021 *	0.015
Focus	0.027 *	0.020	0.029 *	0.018	0.018 *	0.013
007	0.027 *	0.020	0.024 *	0.019	0.019 *	0.012
Penncross	0.022 *	0.017	0.024 *	0.015	0.020 *	0.012
T1	0.024	0.021	0.024 *	0.019	0.021 *	0.014
Tyee	0.025	0.021	0.023 *	0.014	0.019 *	0.014

^a^ Fresh and dry weights were obtained from grass clippings removed at 7, 14 and 21 DPT. ^b^ Phycocyanin (7.6 mg/mL) applied to foliage at 0, 7, 14 and 21 DPT. * Significant difference between treatment and water control based on *t*-tests comparing average fresh and dry clipping weights from 15 replicates over 3 experiments (*p* < 0.05).

**Table 3:** Average shoot and root biomass with weekly foliar application of phycocyanin for different cultivars of creeping bentgrass (*A. stolonifera*) at 28 DPT in the greenhouse.

Cultivar	Shoot				Root			
	Fresh Weight (mg) ^a^		Dry Weight (mg) ^a^		Fresh Weight (mg) ^a^		Dry Weight (mg) ^a^	
	28 DPT		28 DPT		28 DPT		28 DPT	
	Phycocyanin ^b^	Water	Phycocyanin ^b^	Water	Phycocyanin ^b^	Water	Phycocyanin ^b^	Water
Alpha	0.730 *	0.613	0.091 *	0.075	0.982	1.073	0.103	0.105
Focus	0.914 *	0.829	0.095	0.108	1.234	1.236	0.110 *	0.098
007	0.881 *	0.728	0.085	0.097	1.419 *	1.203	0.117 *	0.098
Penncross	0.840 *	0.713	0.115 *	0.097	1.202	1.435	0.119 *	0.099
T1	0.875 *	0.794	0.133 *	0.105	1.096	1.057	0.138 *	0.079
Tyee	0.782 *	0.711	0.083	0.101	1.043 *	1.031	0.094 *	0.087

^a^ Fresh and dry weight shoot and root values were from total plant material harvested at 28 DPT. ^b^ Phycocyanin (7.6 mg/mL) applied to foliage at 0, 7, 14 and 21 DPT. * Significant difference between treatment and water control based on *t*-tests comparing average fresh and dry shoot and root weights from 15 replicates over 3 experiments (*p* < 0.05).

**Table 4:** Average greenness index with weekly foliar application of γ-aminobutyric acid (GABA) for different cultivars of creeping bentgrass (*A. stolonifera*) at 7 to 28 DPT in the greenhouse.

Cultivar	Greenness Index ^a^							
	7 DPT		14 DPT		21 DPT		28 DPT	
	GABA ^b^	Water	GABA	Water	GABA	Water	GABA	Water
Alpha	0.588 *	0.551	0.535	0.535	0.551 *	0.538	0.510	0.527
Focus	0.550	0.549	0.525 *	0.511	0.527 *	0.508	0.489 *	0.480
007	0.546	0.548	0.538 *	0.504	0.538 *	0.512	0.475	0.487
Penncross	0.562	0.559	0.535	0.536	0.532 *	0.521	0.485 *	0.464
T1	0.523	0.542	0.524	0.521	0.539 *	0.509	0.487	0.494
Tyee	0.501	0.507	0.541 *	0.533	0.537 *	0.527	0.485	0.487

^a^ Greenness index values (0 = low to 1 = high) were obtained using FieldScout GreenIndex + Turf Spectrum Technologies, Inc. (Aurora, IL, USA) version 2.0 app. ^b^ GABA (0.016 mg/mL) applied to foliage at 0, 7, 14 and 21 DPT. * Significant difference between treatment and water control based on *t*-tests comparing average greenness index values from 15 replicates over 3 experiments (*p* < 0.05).

**Table 5:** Average clipping biomass with weekly foliar application of γ-aminobutyric acid (GABA) for different cultivars of creeping bentgrass (*A. stolonifera*) at 7 to 21 DPT in the greenhouse.

Cultivar	7 DPT		14 DPT		21 DPT	
	Fresh Weight (mg) ^a^					
	GABA ^b^	Water	GABA	Water	GABA	Water
Alpha	0.045	0.053	0.055	0.056	0.036 *	0.029
Focus	0.059	0.060	0.057	0.057	0.032	0.027
007	0.065 *	0.048	0.053	0.065	0.032	0.034
Penncross	0.061 *	0.053	0.065	0.070	0.033	0.030
T1	0.052	0.064	0.061	0.071	0.043 *	0.036
Tyee	0.057	0.059	0.070 *	0.064	0.034	0.035
**Dry Weight (mg) ^a^**						
Alpha	0.010	0.013	0.015	0.015	0.011 *	0.009
Focus	0.014	0.015	0.015	0.015	0.012 *	0.010
007	0.014	0.013	0.012	0.014	0.012	0.011
Penncross	0.012	0.012	0.014	0.017	0.012 *	0.008
T1	0.014	0.013	0.014	0.016	0.012 *	0.010
Tyee	0.013	0.013	0.017	0.015	0.011	0.012

^a^ Fresh and dry weights were obtained from grass clippings removed at 7, 14 and 21 DPT. ^b^ GABA (0.016 mg/mL) applied to foliage at 0, 7, 14 and 21 DPT. * Significant difference between treatment and water control based on *t*-tests comparing average fresh and dry clipping weights from 15 replicates over 3 experiments (*p* < 0.05).

**Table 6:** Average shoot and root biomass with weekly foliar application of γ-aminobutyric acid (GABA) for different cultivars of creeping bentgrass (*A. stolonifera*) at 28 DPT in the greenhouse.

Cultivar	Shoot				Root			
	Fresh Weight (mg) ^a^		Dry Weight (mg) ^a^		Fresh Weight (mg) ^a^		Dry Weight (mg) ^a^	
	28 DPT		28 DPT		28 DPT		28 DPT	
	GABA ^b^	Water	GABA	Water	GABA	Water	GABA	Water
Alpha	0.599 *	0.509	0.071 *	0.065	1.038 *	0.733	0.089 *	0.069
Focus	0.819 *	0.756	0.093	0.097	1.438 *	1.046	0.104	0.104
007	0.858 *	0.724	0.105 *	0.086	1.443 *	1.067	0.143 *	0.091
Penncross	0.670 *	0.391	0.077 *	0.073	1.465 *	0.758	0.091 *	0.070
T1	0.757 *	0.669	0.087 *	0.067	1.355 *	0.867	0.141 *	0.076
Tyee	0.969 *	0.736	0.060	0.084	1.504 *	0.853	0.070	0.075

^a^ Fresh and dry weight shoot and root values were from total plant material harvested at 28 DPT. ^b^ GABA (0.016 mg/mL) applied to foliage at 0, 7, 14 and 21 DPT. * Significant difference between treatment and water control based on *t*-tests comparing average fresh and dry shoot and root weights from 15 replicates over 3 experiments (*p* < 0.05).

**Table 7:** Average greenness index with weekly foliar application of glycine betaine (GB) for different cultivars of creeping bentgrass (*A. stolonifera*) at 7 to 28 DPT in the greenhouse.

Cultivar	Greenness Index ^a^							
	7 DPT		14 DPT		21 DPT		28 DPT	
	GB ^b^	Water	GB	Water	GB	Water	GB	Water
Alpha	0.582 *	0.551	0.544	0.535	0.555 *	0.538	0.513	0.527
Focus	0.561 *	0.549	0.511	0.511	0.544 *	0.508	0.495 *	0.480
007	0.571 *	0.548	0.527 *	0.504	0.543 *	0.512	0.502 *	0.487
Penncross	0.557	0.559	0.504	0.536	0.542 *	0.521	0.518 *	0.464
T1	0.528	0.542	0.528 *	0.521	0.538 *	0.509	0.500 *	0.494
Tyee	0.530 *	0.507	0.536	0.533	0.547 *	0.527	0.502 *	0.487

^a^ Greenness index values (0 = low to 1 = high) were obtained using FieldScout GreenIndex version 2.0 app. ^b^ GB (0.52 mg/mL) applied to foliage at 0, 7, 14, and 21 DPT. * Significant difference between treatment and water control based on *t*-tests comparing average greenness index values from 15 replicates over 3 experiments (*p* < 0.05).

**Table 8:** Average clipping biomass with weekly foliar application of glycine betaine (GB) for different cultivars of creeping bentgrass (*A. stolonifera*) at 7 to 21 DPT in the greenhouse.

Cultivar	7 DPT		14 DPT		21 DPT	
	Fresh Weight (mg) ^a^					
	GB ^b^	Water	GB	Water	GB	Water
Alpha	0.044	0.053	0.068 *	0.056	0.039 *	0.029
Focus	0.059	0.060	0.057	0.057	0.059 *	0.027
007	0.060 *	0.048	0.084 *	0.065	0.050 *	0.034
Penncross	0.058	0.056	0.065	0.070	0.044 *	0.030
T1	0.053	0.061	0.080 *	0.071	0.045 *	0.036
Tyee	0.057	0.059	0.070 *	0.064	0.060 *	0.035
	**Dry Weight (mg) ^a^**					
Alpha	0.013	0.013	0.013	0.015	0.013 *	0.009
Focus	0.013	0.015	0.015	0.015	0.017 *	0.010
007	0.014	0.013	0.016 *	0.014	0.016 *	0.011
Penncross	0.013	0.012	0.014	0.017	0.015 *	0.008
T1	0.014	0.013	0.016	0.016	0.013	0.010
Tyee	0.013	0.013	0.017 *	0.015	0.017 *	0.012

^a^ Fresh and dry weights were obtained from grass clippings removed at 7, 14 and 21 DPT. ^b^ GB (0.52 mg/mL) applied to foliage at 0, 7, 14, and 21 DPT. * Significant difference between treatment and water control based on *t*-tests comparing average fresh and dry clipping weights from 15 replicates over 3 experiments (*p* < 0.05).

**Table 9:** Average shoot and root biomass with weekly foliar application of glycine betaine (GB) for different cultivars of creeping bentgrass (*A. stolonifera*) at 28 DPT in the greenhouse.

Cultivar	Shoot				Root			
	Fresh Weight (mg) ^a^		Dry Weight (mg) ^a^		Fresh Weight (mg) ^a^		Dry Weight (mg) ^a^	
	28 DPT		28 DPT		28 DPT		28 DPT	
	GB ^b^	Water	GB	Water	GB ^b^	Water	GB	Water
Alpha	0.784 *	0.509	0.072 *	0.065	0.968 *	0.733	0.013 *	0.009
Focus	0.915 *	0.756	0.089	0.097	1.324 *	1.046	0.017 *	0.010
007	1.002 *	0.724	0.092 *	0.086	1.195 *	1.067	0.016 *	0.011
Penncross	0.810 *	0.540	0.086 *	0.073	1.087 *	0.758	0.015 *	0.008
T1	0.915 *	0.669	0.083 *	0.067	1.157 *	0.867	0.013 *	0.010
Tyee	1.041 *	0.736	0.105 *	0.084	1.371 *	0.853	0.017 *	0.012

^a^ Fresh and dry weight shoot and root values were from total plant material harvested at 28 DPT. ^b^ GB (0.52 mg/mL) applied to foliage at 0, 7, 14, and 21 DPT. * Significant difference between treatment and water control based on *t*-tests comparing average fresh and dry shoot and root weights from 15 replicates over 3 experiments (*p* < 0.05).

**Table 10:** Average greenness index with weekly foliar application of *Rhizophagus intraradices* (MYKE PRO) for different cultivars of creeping bentgrass (*A. stolonifera*) at 7 to 28 DPT in the greenhouse.

Cultivar	Greenness Index ^a^							
	7 DPT		14 DPT		21 DPT		28 DPT	
	MYKE PRO ^b^	Water	MYKE PRO	Water	MYKE PRO	Water	MYKE PRO	Water
Alpha	0.580 *	0.566	0.577 *	0.568	0.485	0.490	0.515 *	0.504
Focus	0.570 *	0.545	0.551	0.551	0.506	0.510	0.499	0.516
007	0.562	0.560	0.563 *	0.556	0.501	0.506	0.497	0.498
Penncross	0.572 *	0.562	0.556 *	0.550	0.492	0.491	0.498 *	0.491
T1	0.577 *	0.537	0.557	0.558	0.516 *	0.502	0.506 *	0.502
Tyee	0.566 *	0.554	0.550	0.565	0.508 *	0.493	0.515 *	0.505

^a^ Greenness index values (0 = low to 1 = high) were obtained using FieldScout GreenIndex version 2.0 app. ^b^ MYKE PRO (0.30 mg/mL) applied to foliage at 0, 7, 14, and 21 DPT. * Significant difference between treatment and water control based on *t*-tests comparing average greenness index values from 15 replicates over 3 experiments (*p* < 0.05).

**Table 11:** Average clipping biomass with weekly foliar application of *Rhizophagus intraradices* (MYKE PRO) for different cultivars of creeping bentgrass (*A. stolonifera*) at 7 to 21 DPT in the greenhouse.

Cultivar	7 DPT		14 DPT		21 DPT	
	Fresh Weight (mg) ^a^					
	MYKE PRO ^b^	Water	MYKE PRO	Water	MYKE PRO	Water
Alpha	0.126 *	0.096	0.073	0.085	0.022	0.035
Focus	0.095	0.110	0.105	0.105	0.029	0.034
007	0.139 *	0.100	0.093 *	0.088	0.023	0.028
Penncross	0.095 *	0.080	0.088	0.084	0.022	0.026
T1	0.106	0.126	0.110	0.112	0.027	0.035
Tyee	0.074	0.107	0.084	0.088	0.029 *	0.019
	**Dry Weight (mg) ^a^**					
Alpha	0.023 *	0.021	0.019 *	0.017	0.010	0.015
Focus	0.022 *	0.020	0.020	0.020	0.012	0.012
007	0.027 *	0.020	0.023 *	0.019	0.009	0.010
Penncross	0.021 *	0.017	0.019 *	0.017	0.009	0.010
T1	0.022 *	0.020	0.024 *	0.021	0.012	0.012
Tyee	0.021	0.021	0.017	0.016	0.008	0.013

^a^ Fresh and dry weights were obtained from grass clippings removed at 7, 14 and 21 DPT. ^b^ MYKE PRO (0.30 mg/mL) applied to foliage at 0, 7, 14, and 21 DPT. * Significant difference between treatment and water control based on *t*-tests comparing average fresh and dry clipping weights from 15 replicates over 3 experiments (*p* < 0.05).

**Table 12:** Average shoot and root biomass with weekly foliar application of *Rhizophagus intraradices* (MYKE PRO) for different cultivars of creeping bentgrass (*A. stolonifera*) at 28 DPT in the greenhouse.

Cultivar	Shoot				Root			
	Fresh Weight (mg) ^a^		Dry Weight (mg) ^a^		Fresh Weight (mg) ^a^		Dry Weight (mg) ^a^	
	28 DPT		28 DPT		28 DPT		28 DPT	
	MYKE PRO ^b^	Water	MYKE PRO	Water	MYKE PRO	Water	MYKE PRO	Water
Alpha	0.663	0.706	0.094 *	0.081	1.079	1.151	0.134 *	0.100
Focus	0.839	0.967	0.142 *	0.118	1.172	1.587	0.110	0.110
007	0.781	0.826	0.126 *	0.105	1.332	1.515	0.131 *	0.107
Penncross	0.648	0.819	0.107	0.109	1.081	1.651	0.107	0.102
T1	0.714	0.916	0.104	0.117	0.921	1.183	0.138 *	0.090
Tyee	0.656	0.822	0.091	0.114	0.850	1.213	0.102 *	0.095

^a^ Fresh and dry weight shoot and root values were from total plant material harvested at 28 DPT. ^b^ MYKE PRO (0.30 mg/mL) applied to foliage at 0, 7, 14, and 21 DPT. * Significant difference between treatment and water control based on *t*-tests comparing average fresh and dry shoot and root weights from 15 replicates over 3 experiments (*p* < 0.05).

## Data Availability

The data that support the findings of this study are available on request from the corresponding author (P.H.G). (Email address: pgoodwin@uoguelph.ca). The data are not publicly available due to their being used in an ongoing project.

## Abbreviations

The following abbreviations are used in this manuscript:

ALA	5-aminolevulinic acid
ANOVA	analysis of variance
AMF	arbuscular mycorrhizal fungus
DGCI	dark green color index
DPT	days post treatment
GABA	γ-aminobutyric acid
GB	glycine betaine
GTI	Guelph Turfgrass Institute
MYKE PRO	*Rhizophagus intraradices*
PROC GLM	general linear model procedure
RGB	red, green, and blue
